# Nucleosome organization in the vicinity of transcription factor binding sites in the human genome

**DOI:** 10.1186/1471-2164-15-493

**Published:** 2014-06-19

**Authors:** Yumin Nie, Xiangfei Cheng, Jiao Chen, Xiao Sun

**Affiliations:** State Key Laboratory of Bioelectronics, School of Biological Science & Medical Engineering, Southeast University, 210096 Nanjing, China

**Keywords:** Nucleosome occupancy, Transcription factor binding site, Clustering

## Abstract

**Background:**

The binding of transcription factors (TFs) to specific DNA sequences is an initial and crucial step of transcription. In eukaryotes, this process is highly dependent on the local chromatin state, which can be modified by recruiting chromatin remodelers. However, previous studies have focused mainly on nucleosome occupancy around the TF binding sites (TFBSs) of a few specific TFs. Here, we investigated the nucleosome occupancy profiles around computationally inferred binding sites, based on 519 TF binding motifs, in human GM12878 and K562 cells.

**Results:**

Although high nucleosome occupancy is intrinsically encoded at TFBSs *in vitro*, nucleosomes are generally depleted at TFBSs *in vivo*, and approximately a quarter of TFBSs showed well-positioned *in vivo* nucleosomes on both sides. RNA polymerase near the transcription start site (TSS) has a large effect on the nucleosome occupancy distribution around the binding sites located within one kilobase to the nearest TSS; fuzzier nucleosome positioning was thus observed around these sites. In addition, in contrast to yeast, repressors, rather than activators, were more likely to bind to nucleosomal DNA in the human cells, and nucleosomes around repressor sites were better positioned *in vivo*. Genes with repressor sites exhibiting well-positioned nucleosomes on both sides, and genes with activator sites occupied by nucleosomes had significantly lower expression, suggesting that actions of activators and repressors are associated with the nucleosome occupancy around their binding sites. It was also interesting to note that most of the binding sites, which were not in the DNase I-hypersensitive regions, were cell-type specific, and higher *in vivo* nucleosome occupancy were observed at these binding sites.

**Conclusions:**

This study demonstrated that RNA polymerase and the functions of bound TFs affected the local nucleosome occupancy around TFBSs, and nucleosome occupancy patterns around TFBSs were associated with the expression levels of target genes.

**Electronic supplementary material:**

The online version of this article (doi: 10.1186/1471-2164-15-493) contains supplementary material, which is available to authorized users.

## Background

Transcription factors (TFs) bind to specific DNA sequences and interact with components of the RNA polymerase complex, or with other complexes, to regulate transcription in a cell type-specific manner, and this process is highly dependent on the chromatin structure in eukaryotes [1–3]. The basic unit of chromatin structure is the nucleosome, consisting of histone octamers wrapped in 147 base pairs (bps) of DNA [4, 5]. Eukaryotic genomic DNA is assembled into nucleosomes and is further packaged into chromatin to achieve high compaction. Nucleosomes can directly regulate the accessibility of TFs and transcriptional machinery to the DNA sequences [6]. Sequences in nucleosome-depleted regions are easier to access, while the accessibility of DNA within nucleosomes depends on nucleosome dynamics [7, 8]. Although histone-DNA complexes are very stable, histones are constantly evicted and reassembled onto DNA templates in a locus-specific manner. Previous studies have suggested that promoters and other regulatory sequences are typically nucleosome-depleted, whereas transcribed regions tend to be occupied by well-positioned nucleosomes, which are maintained by nucleosome-remodeling activities [9, 10]. The occupancy patterns and dynamic positioning of nucleosomes thus play crucial roles in regulating eukaryotic transcription.

Nucleosomes influence the accessibility of TFs to DNA. TFs can, in turn, directly or indirectly recruit remodeling complexes, or other coregulators, to modify the local chromatin state. The binding of several TFs, such as the insulator binding protein CTCF [11, 12], the RE1-silencing transcription factor (REST/NRSF) [13] and the multifunctional TF YY1 [14], has been suggested to initiate nucleosome depletion at TF binding sites (TFBSs) and the phased nucleosome arrays in the flanking regions in human cells. Nearly 3,000 TFs have been predicted computationally in the DNA-binding domain (DBD) database [15], and detailed manual curation has confirmed at least 1,400 TFs in the human genome [16]. However, chromatin immunoprecipitation followed by sequencing (ChIP-seq), a technique for measuring genome-wide TF binding profiles, is only applied to one TF in a single experiment, making it difficult to identify binding locations for large numbers of factors in the specific cell type. Previous studies have, therefore, focused mainly on nucleosome occupancy around binding sites of a few specific TFs [11–13, 17]. Computational methods have the advantage of being able to determine the accurate profiles for many factors in a specific sample [18, 19]. Like many computational methods, CENTIPEDE [19], based on a hierarchical Bayesian mixture model, requires position weight matrices of known TF binding motifs; therefore, its ability is dependent on the availability of TF binding motifs. However, CENTIPEDE incorporates cell-specific experimental data to infer binding sites in a particular cell type, making it more accurate for predicting TFBSs.

To better understand the relationship between nucleosome positioning and TF binding, we focused on the CENTIPEDE-inferred binding sites for 519 TF binding motifs, representing up to a third of the human TF repertoire, and examined the nucleosome occupancy around these binding sites in human GM12878 and K562 cells. We further classified the binding sites by the distances of sites relative to the nearest gene and the functions of the bound TFs, to test whether the nucleosome occupancy exhibited distinct patterns. We finally clustered the nucleosome occupancy profiles around TFBSs and investigated their relevance to gene expression.

## Results

### Nucleosome occupancy around TF binding sites

**Figure 1:**
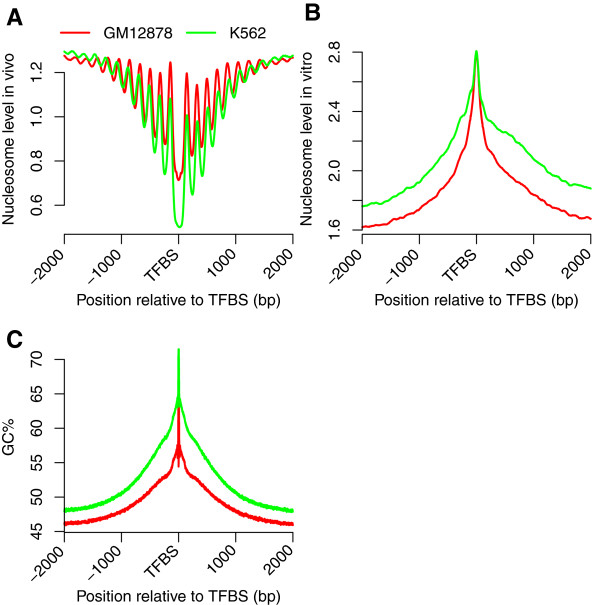
**Nucleosome occupancy profiles and GC content around TF binding sites. (A)** Nucleosomes were depleted at TFBSs and well positioned in the flanking regions *in vivo*. **(B)** High *in vitro* nucleosome occupancy was encoded at TFBSs. **(C)** High GC content was observed at TFBSs.

Both *in vitro* and *in vivo* nucleosome occupancy data were used to examine the average nucleosome occupancy around the binding sites for 519 TF binding motifs (Additional file 1) in GM12878 and K562 cells. Nucleosomes can be assembled by genomic DNA and recombinant histones in the absence of cellular influences; therefore, *in vitro* nucleosome occupancy is affected mainly by the intrinsic specificity between a histone and the DNA sequences, whereas *in vivo* occupancy is influenced by sequence preferences, TFs and chromatin remodelers [5, 13, 20]. Analyses of *in vivo* data showed nucleosome-depleted regions at TFBSs and an array of well-positioned nucleosomes in the flanking regions (Figure 1A), which was consistent with the barrier model suggested in previous studies [4, 13]. We then constructed *in vitro* nucleosome occupancy profiles, to test whether the *in vivo* nucleosome distributions were governed by the intrinsic sequence preferences of nucleosomes. We observed high *in vitro* nucleosome occupancy at TFBSs (Figure 1B), suggesting that binding sequences of TFs tend to form nucleosomes. We further analyzed the nucleotide composition of TF binding sequences and found that these DNA sequences were GC-rich (Figure 1C). Our analyses, which are consistent with previous studies [13, 17], demonstrated that human TFBSs have high GC content and intrinsic nucleosome occupancy, but low *in vivo* nucleosome occupancy.

Low nucleosome signals *in vivo* are necessary for most TFBSs, as TFs may compete with nucleosomes to slide or evict them for access to the specific DNA [8]. It is generally believed that nucleosomes are depleted before TFs bind to their sites. However, a recent study argues that nucleosome eviction occurs after TF binding and, in fact, requires TF binding, suggesting that nucleosome loss may not be a prerequisite for TF binding [21]. The barrier model could explain the well-positioned nucleosomes around TFBSs. Binding of TFs can form barriers and other nucleosomes are stacked against them to generate the phased nucleosome arrays by ATP-dependent chromatin remodelers [4, 10]. Despite DNA sequences encoding nucleosome occupancy at certain regions, TF binding can drive nucleosomes to occupy intrinsically unfavorable DNA sequences or evict nucleosomes from intrinsically favorable sites.

### Nucleosome occupancy around proximal and distal binding sites

**Figure 2:**
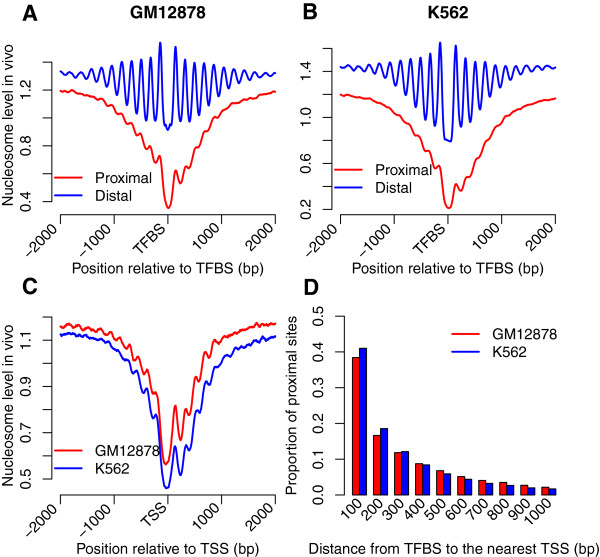
***In vivo*** **nucleosome occupancy profiles around proximal sites are similar with those around TSSs. (A, B)** *In vivo* nucleosome occupancy profiles around the proximal and distal sites in both GM12878 and K562 cells. **(C)** *In vivo* nucleosome occupancy profiles around TSSs. **(D)** Proportion of proximal sites in each 100-bp interval.

TFBSs may be located proximal or distal to the transcription start site (TSS). Binding sites in the core or proximal promoter are typically located within one kilobase (kb), while distal sites may be situated up to several hundred kb from the core promoter [22]. Here, we defined proximal and distal sites as those located within 1 kb and beyond 10 kb from the nearest TSS, respectively, and investigated the *in vivo* nucleosome occupancy around these sites. The nucleosome occupancy profiles around distal sites in both GM12878 and K562 cells (Figure 2A and B) were similar to those around all binding sites, as described in Figure 1A. However, we observed lower *in vivo* nucleosome occupancy and fuzzier nucleosome positioning around proximal sites, which were more consistent with those around TSSs (Figure 2C). Nucleosome-depleted regions are also essential for TSSs, as RNA polymerase and a variety of auxiliary components bind to DNA and interact with each other around TSSs [4]. In order to explain the *in vivo* nucleosome occupancy pattern around the proximal sites, we divided proximal sites into 100-bp intervals based on their distances relative to the nearest TSS, and investigated the proportion of proximal sites in each interval. Our results indicated that 55.1% and 59.6% of proximal sites in GM12878 and K562 cells, respectively, were located within 200 bp relative to TSSs (Figure 2D), suggesting that many proximal sites are located within nucleosome-depleted regions near TSSs, and nucleosome occupancy around proximal sites thus mostly reflect that around TSSs. Proximal sites fall within promoter regions near TSSs, where nucleosomes are generally depleted, and therefore the average nucleosome occupancy is lower at proximal sites and the nucleosome positioning around proximal sites is less pronounced. On the other hand, distal sites are far from promoters, where the nucleosome occupancy is higher, the binding of TFs in the distal regions is therefore more likely to recruit ATP-dependent chromatin remodelers to generate the phased nucleosome arrays.

### Nucleosome occupancy around activator and repressor binding sites

**Figure 3:**
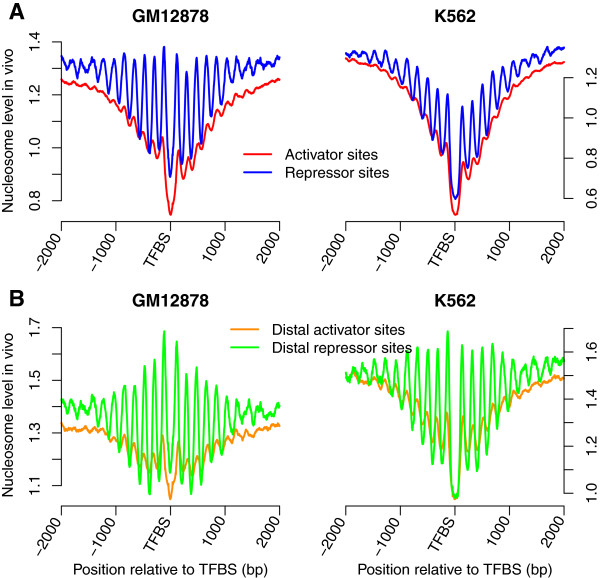
***In vivo*** **, nucleosomes are better positioned around repressor sites. (A)** *In vivo* nucleosome occupancy profiles around the activator and repressor sites in both GM12878 and K562 cells. **(B)** *In vivo* nucleosome occupancy profiles around the distal activator and repressor sites.

**Figure 4:**
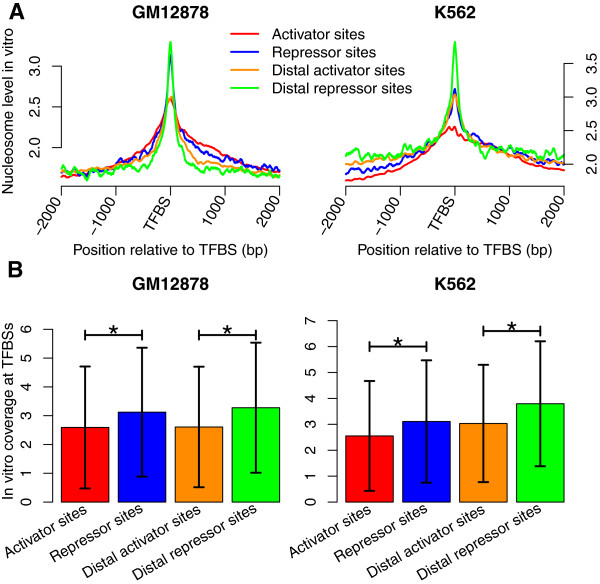
**Repressor binding sequences are more likely to encode nucleosomes intrinsically. (A)** *In vitro* nucleosome occupancy around the activator and repressor sites, and their distal sites in both GM12878 and K562 cells. **(B)** Average *in vitro* nucleosome occupancy at the activator and repressor sites, and their distal sites. The *in vitro* nucleosome signals were normalized by the length of TFBSs and given as the mean ± standard deviation. Statistically significant differences were detected using a Wilcoxon rank-sum test. (*) *P* < 2.2 × 10^−16^.

Activators and repressors positively and negatively regulate transcription, respectively. Both activators, such as Abf1 [23] and Reb1 [24] in yeast, and repressors, such as the REST protein [13] in human, have been suggested to bind to DNA sequences that intrinsically encode high nucleosome occupancy, and their binding will influence the nucleosome organization. In yeast, it has also been suggested that activator binding sites show significantly higher correlation with nucleosome sequence profiles compared with those of repressors [25]. Here, we queried the UniProt database to determine the functions of TFs [26], and investigated the nucleosome occupancy around activator and repressor binding sites to test whether the functions of bound TFs influenced the nucleosome occupancy distribution in human cells. Activators and repressors identified in GM12878 and K562 cells were listed in Additional file 2. We first examined the *in vivo* nucleosome occupancy around activator and repressor binding sites. Although nucleosome-depleted regions were observed at activator and repressor binding sites, nucleosomes around the repressors sites were better positioned compared with the activator sites in both GM12878 and K562 cells (Figure 3A). Repressor binding sites are generally situated quite distally from TSSs, while multiple binding sites for activators are located in the promoter [22]. 55.0% of repressor sites and 44.9% of activators sites were located beyond 10 kb from TSS in the GM12878 cell line, while in the K562 cell line, the percentages of distal repressor and activator sites were 36.5% and 28.9%, respectively. Therefore, we examined the *in vivo* nucleosome occupancy around the distal activator and repressor sites (Additional file 3) to avoid the influences of the transcriptional machinery near TSSs. Better-positioned nucleosomes were observed around the distal repressor sites (Figure 3B), indicating that repressor binding induced the nucleosome distribution. We further investigated the *in vivo* nucleosome occupancy around the distal sites for each of activators and repressors. Both activators, such as FLI1 and NFE2, and repressors, such as REST and Tel-2, can generate the phased nucleosome array (Additional file 4 and Additional file 5). However, the nucleosome positioning around REST binding sites was particularly obvious, which contributed largely to the better nucleosome positioning around repressor sites. It should be noted that for some repressors, such as Bcl6b_2, fuzzy nucleosome positioning was observed around their binding sites, suggesting that the formation of phased nucleosome array is associated with the specific TFs. We finally examined the *in vitro* nucleosome occupancy around activator and repressor binding sites. Both activator and repressor sites were enriched in nucleosome sequence preferences (Figure 4A). However, unlike yeast, repressor binding sequences were more likely to intrinsically encode nucleosomes in the human genome, according to the analysis of the *in vitro* data (Figure 4B; Wilcoxon rank-sum test, *P* < 2.2 × 10^−16^). The same conclusion was obtained from the analyses of distal sites. Repressor binding to sequences with higher *in vitro* nucleosome occupancy might have more elevated dependence on nucleosome dynamics and chromatin remodeling complexes [27, 28], and this might contribute to the better-positioned nucleosomes flanking the repressor sites *in vivo*.

### Nucleosome occupancy around DNase I-hypersensitive and -resistant sites

**Figure 5:**
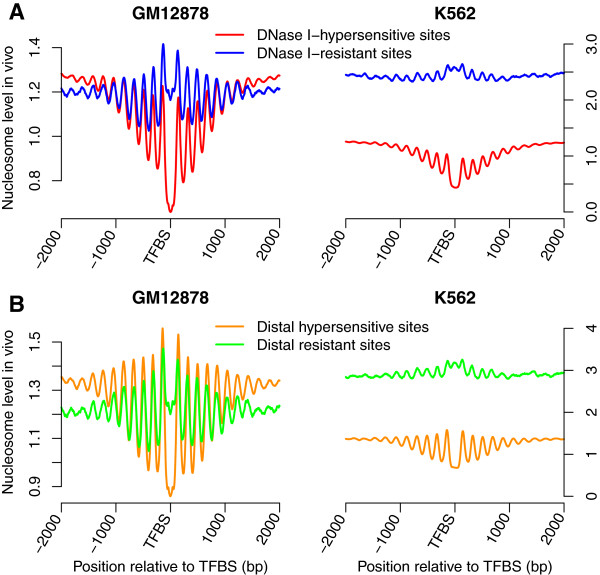
**DNase I-resistant binding sites show higher** ***in vivo*** **nucleosome occupancy. (A)** *In vivo* nucleosome occupancy around DNase I-hypersensitive and -resistant binding sites in both GM12878 and K562 cells. **(B)** *In vivo* nucleosome occupancy around distal DNase I-hypersensitive and -resistant binding sites.

**Figure 6:**
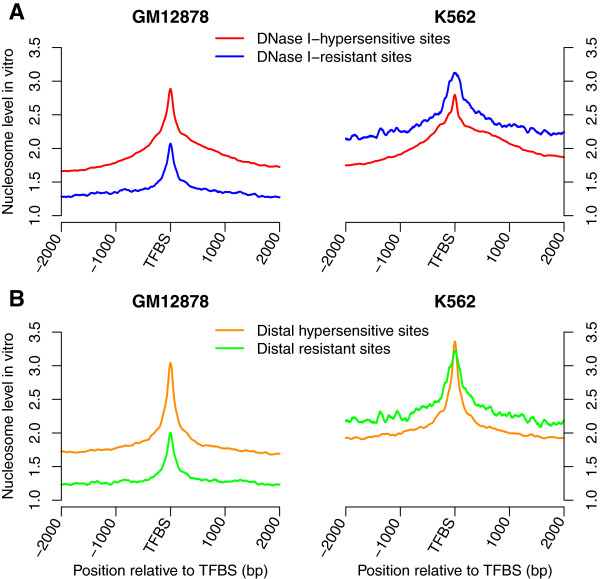
**DNase I-resistant binding regions are more likely to form nucleosomes in the K562 cell line. (A)** *In vitro* nucleosome occupancy around DNase I-hypersensitive and -resistant sites in both GM12878 and K562 cells. **(B)** *In vitro* nucleosome occupancy around distal DNase I-hypersensitive and -resistant sites.

TFs compete with nucleosomes to access DNA and nucleosome depletion is thus generally observed at their binding sites. However, TFs can bind to DNA sequences with high *in vivo* nucleosome occupancy [5]. DNase I preferentially digests DNA in regions of low nucleosome occupancy and DNase I-hypersensitive regions reflect the accessibility of genome. We found that 10.8% and 3.1% of the binding sites in GM12878 and K562 cells, respectively, were not in DNase I-hypersensitive regions, and observed higher *in vivo* nucleosome occupancy at these DNase I-resistant binding sites, especially in the K562 cell line (Figure 5). We then investigated the *in vitro* nucleosome occupancy around the DNase I-hypersensitive and -resistant sites. Similar *in vitro* nucleosome occupancy levels were observed around the DNase I-hypersensitive sites in GM12878 and K562 cells. However, for DNase I-resistant binding sites, *in vitro* nucleosome occupancy level in the K562 cell line was higher than that in the GM12878 cell line (Figure 6A). The same conclusions were obtained by analyzing the distal DNase I-hypersensitive and -resistant sites (Figure 6B). DNA sequences around DNase I-resistant sites in the K562 cell line were more likely to form nucleosomes, and TF binding in the DNase I-resistant regions had less effect on the local chromatin structure, which might contribute to the higher *in vivo* nucleosome occupancy around DNase I-resistant sites in the K562 cell line. CTCF, serum response factor SRF and c-Rel were three TFs that most frequently bound to DNase I-resistant regions in GM12878 cells, whereas the three TFs that most frequently bound to these regions were CTCF, SRF and HNF4 in K562 cells. CTCF is a multifunctional TF, while SRF and c-Rel are considered as activators. It was also interesting to note that most of the DNase I-resistant binding sites were cell-type specific. 90.5% and 81.5% of these sites in GM12878 and K562 cells, respectively, were cell-type specific and were not bound by any TF in the other type of cells. These results suggested that the DNase I-resistant sites might play important roles in the specific cells.

### Clustering nucleosome occupancy around TF binding sites

**Figure 7:**
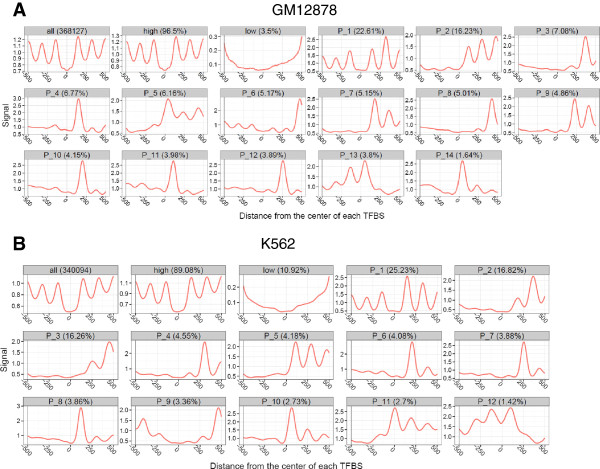
**Clustering nucleosome signals around all TF binding sites. (A)** Nucleosome occupancy clusters identified using CAGT in the GM12878 cell line. The first panel was a traditional aggregate plot of the nucleosome occupancy signals for all TFBSs. The second and third panels were traditional aggregate plots for TFBSs with high and low nucleosome signals. The rest of the panels exhibited the nucleosome signals averaged over the sites in each of the clusters, and the proportion of binding sites in each cluster was given in the header. **(B)** Nucleosome occupancy clusters identified using CAGT in the K562 cell line.

Some TFs can bind to the DNase I-resistant regions with high *in vivo* nucleosome occupancy and might regulate gene expression in a cell-type-specific way. A traditional profile that averages over all TF binding sites will neglect these specific nucleosome occupancy patterns. Therefore we applied an unsupervised clustering method called Clustered AGgregation Tool (CAGT) [29], to discover the diverse *in vivo* nucleosome occupancy patterns around TFBSs. The cluster analysis of all TFBSs indicated that only 22.6% and 25.2% of TFBSs in GM12878 and K562 cells, respectively, were flanked by well-positioned nucleosomes on both sides (Figure 7). The cluster analysis of proximal and distal binding sites further indicated that TFBSs exhibiting symmetric positioning of nucleosomes were mainly located in the distal regions (Additional file 6). We clustered the nucleosome signals around distal activator and repressor binding sites and found that higher proportions of distal repressors sites showed strong nucleosome positioning on both sides (Additional file 7). All these results were consistent with our previous analyses. However, the asymmetric nucleosome occupancy patterns were neglected in our previous analyses. The cluster analysis of nucleosome signals around TFBSs indicated that the majority of TFBSs showed strong nucleosome positioning on one side, suggesting that asymmetric patterns of nucleosome occupancy were more pervasive around TFBSs.

### Nucleosome occupancy patterns correlate with gene expression

**Figure 8:**
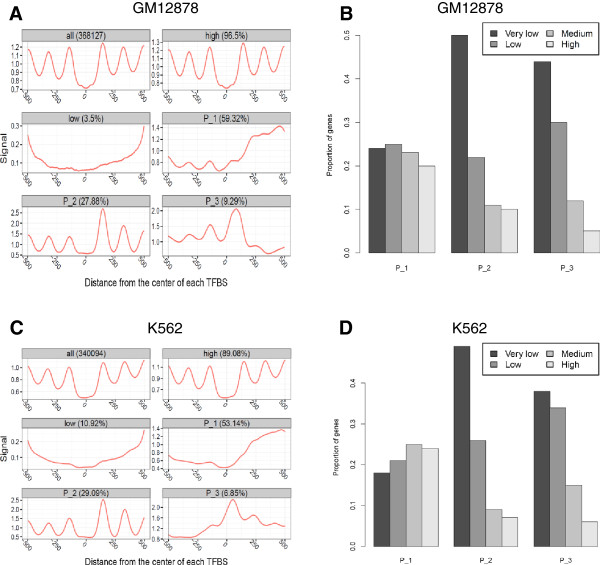
**Three nucleosome occupancy clusters around all TF binding sites and expression levels of target genes for each of the clusters. (A)** Three nucleosome occupancy clusters identified using CAGT in the GM12878 cell line. The first three panels were traditional aggregate plots of the nucleosome occupancy signals for all TFBSs, TFBSs with high nucleosome signals and TFBSs with low nucleosome signals. The rest of the panels exhibited three nucleosome occupancy patterns averaged over the sites in each of the clusters. **(B)** Expression levels of target genes for each of the clusters in the GM12878 cell line. Target genes were divided into four categories based on their expression levels: very lowly, lowly, medium and highly expressed genes. The *y* axis indicated the proportions of target genes that fell into each category. **(C)** Three nucleosome occupancy clusters identified using CAGT in the K562 cell line. **(D)** Expression levels of target genes for each of the clusters in the K562 cell line.

It has been suggested that nucleosome occupancy patterns around TSSs are associated with gene expression [29, 30]. Here we assigned each of the binding sites to the nearest gene based on its distance to TSSs [31, 32], and investigated the correlations between *in vivo* nucleosome occupancy patterns around TFBSs and gene expression. First, we grouped the nucleosome occupancy signals of all TFBSs into three clusters using the CAGT software, and examined the expression levels of unique target genes for each of the clusters in both GM12878 and K562 cells (Figure 8). Increasing the number of clusters would lead to a decrease in the number of target genes for each cluster, and we empirically grouped into three clusters. TFBSs in the first cluster in both GM12878 and K562 cells showed fuzzy nucleosome positioning and relatively higher nucleosome occupancy on one side (P_1); TFBSs in the second cluster had strongly positioned nucleosomes on both sides (P_2); and TFBSs in the third cluster were occupied by nucleosomes (P_3). Analysis of gene expression levels for each of the clusters in both GM12878 and K562 cells indicated that TFBSs in the first cluster were associated with genes that were in general significantly more highly expressed (Figure 8B and D; Wilcoxon rank-sum test, *P* < 3.7 × 10^−16^).

**Figure 9:**
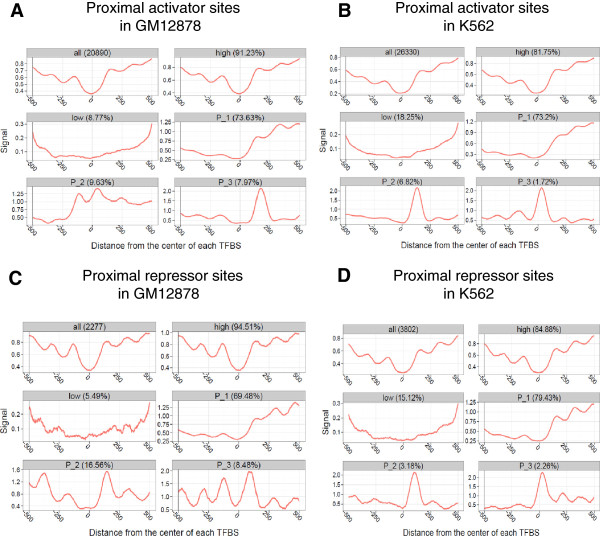
**Three nucleosome occupancy clusters around proximal activator and repressor binding sites. (A)** Three nucleosome occupancy clusters for proximal activator binding sites in the GM12878 cell line. The first three panels were traditional aggregate plots of the nucleosome occupancy signals for all proximal activator sites, sites with high nucleosome signals and sites with low nucleosome signals. The rest of the panels exhibited three nucleosome occupancy patterns averaged over the proximal activator sites in each of the clusters. **(B)** Three nucleosome occupancy clusters for proximal activator binding sites in the K562 cell line. **(C, D)** Three nucleosome occupancy clusters for proximal repressor binding sites in GM12878 and K562 cells.

**Figure 10:**
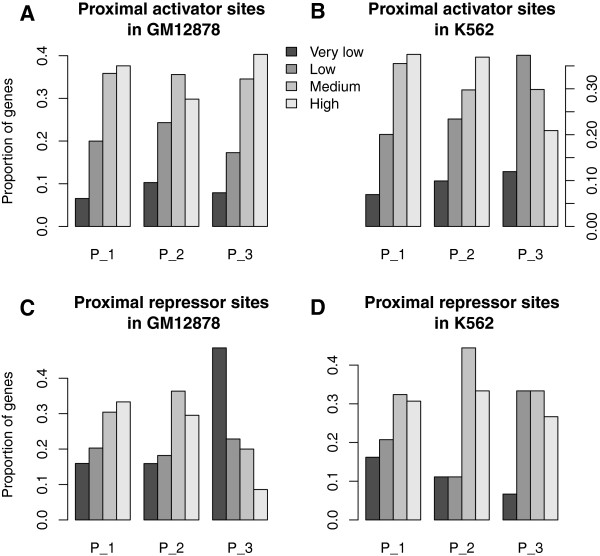
**Expression levels of target genes for each cluster of proximal activator and repressor binding sites. (A)** Expression levels of target genes for each cluster of proximal activator binding sites in the GM12878 cell line. Genes associated with binding sites in Pattern 2 had significantly lower expression. **(B)** Expression levels of target genes for each cluster of proximal activator binding sites in the K562 cell line. Genes associated with sites in Pattern 3 had significantly lower expression. **(C)** Expression levels of target genes for each cluster of proximal repressor binding sites in the GM12878 cell line. Genes associated with proximal repressor sites in Pattern 3 had significantly lower expression. **(D)** Expression levels of target genes for each cluster of proximal repressor binding sites in the K562 cell line. Statistically significant differences were detected using a Wilcoxon rank-sum test.

Activators and repressors have the opposite effects on gene expression, but the binding of both of them can generate similar patterns of nucleosome occupancy (Additional file 7). In order to clearly demonstrate the correlations between TF binding and gene expression, we further clustered the nucleosome occupancy signals around activator and repressor binding sites using the CAGT software, and examined their relevance to gene expression in both GM12878 and K562 cells. Nucleosome signals around proximal activator binding sites in both GM12878 and K562 cells were first grouped into three clusters using the CAGT software (Figure 9A and B). Pattern 1 (P_1) in both GM12878 and K562 cells showed fuzzy nucleosome positioning and higher nucleosome occupancy on one side of TFBSs; Pattern 2 (P_2) in GM12878 and Pattern 3 (P_3) in K562 showed high nucleosome occupancy at TFBSs; Pattern 3 (P_3) in GM12878 and Pattern 2 (P_2) in K562 exhibited one well-positioned nucleosome on one side of TFBSs. The analysis of target gene expression for each cluster of proximal activator sites indicated that genes associated with proximal activator sites in Pattern 2 had significantly lower expression in the GM12878 cell line (Figure 10A; Wilcoxon rank-sum test, *P* < 0.002), and genes associated with sites in Pattern 3 had significantly lower expression in the K562 cell line (Figure 10B; *P* < 0.007). The same analysis for the proximal repressor binding sites indicated that genes associated with proximal repressor sites in Pattern 3 (P_3) had significantly lower expression in the GM12878 cell line (Figure 10C; Wilcoxon rank-sum test, *P* < 0.0006). Pattern 3 in GM12878 showed well-positioned nucleosomes on both sides of proximal repressor sites (Figure 9C). We observed no significant differences in the expression levels of target genes for different clusters of proximal repressor sites in the K562 cell line (Figure 9D and Figure 10D). We also performed the same analyses on the distal activator and repressor binding sites in both GM12878 and K562 cells, and found no significant differences in the expression levels of target genes for different clusters of distal activator and repressor sites (Additional file 8 and Additional file 9). This might result from the assignment of target genes. We simply assigned target genes using the nearest distance, and it would be unreliable for the binding sites distal to TSSs.

## Discussion

Transcription is regulated by the dynamic binding of TFs to the underlying DNA sequences in a cell type-specific manner [3]. Most eukaryotic genomic DNA is packaged into nucleosomes, and TF binding is thus strongly associated with the local chromatin structure surrounding TFBSs [4, 5]. Chromatin can affect the recognition and binding of TFs; TFs can, in turn, direct the chromatin remodeling complexes to their target regions [2]. We examined the nucleosome occupancy profiles around TFBSs to better understand the intricate relationships between TF binding and chromatin structure, and we also investigated the correlations between binding sites with different patterns of nucleosome occupancy and gene expression.

Although previous studies have demonstrated the nucleosome occupancy profiles around the binding sites for several specific TFs, our findings expand the current knowledge of nucleosome occupancy at TFBSs, based on the greater number of TFs. First, TF binding regions are generally nucleosome-depleted as a result of TF and nucleosome interactions. TFs can directly compete with nucleosomes and evict them from the DNA, while some TFs are aided by pioneer factors to bind to DNA [6–8]. Pioneer factors, such as FoxA, GATA, and PU.1, can bind to nucleosomal DNA and displace nucleosomes to help other TFs access their sites [33]. Although nucleosome-depleted regions are necessary for most TFBSs, some TFs may bind to nucleosomal DNA without nucleosome reorganization. Previous studies have suggested that TF NF-κB p50 can bind to nucleosomal DNA without perturbing the overall structure of the nucleosome [34]. We found that many TFBSs were located within DNase-I resistant regions, and these DNase-I resistant sites were cell-type specific. The cluster analysis using the CAGT software also indicated that a small proportion of TFBSs were indeed occupied by nucleosomes. It should be also noted that TFs might directly bind to sites in the nucleosome-depleted regions, especially in the proximal promoter near TSSs. Second, better-positioned nucleosomes were observed around the repressor sites compared with those around the activator sites. Repressors were more likely to bind to nucleosomal DNA, which might require catalyzed remodeling, in the human genome. The higher dependence on chromatin remodeling complexes might contribute to the stronger nucleosome positioning around the repressor sites. Besides, repressors are more associated with closed chromatin compared with activators. The highly positioned nucleosomes might result from the recruitment of different chromatin remodelers. Third, although a quarter of TFBSs showed arrays of well-positioned nucleosomes on both sides, the majority of TFBSs exhibited one or more well-positioned nucleosomes on one side, and a small proportion of TFBSs were occupied by nucleosomes *in vivo*. Correlating these different patterns of nucleosome occupancy with the expression levels of target genes indicated that genes with TFBSs exhibiting well-positioned nucleosomes on both sides or occupied by nucleosomes, had significantly lower expression levels. The analysis of gene expression for proximal activator and repressor binding sites further indicated that genes with repressor sites exhibiting well-positioned nucleosomes on both sides, and genes with activator sites occupied by nucleosomes had significantly lower expression, suggesting that actions of activators and repressors are associated with the nucleosome occupancy around their binding sites.

## Conclusions

The DNA sequence, TF binding and chromatin remodeling events are important determinants of *in vivo* nucleosome organization in human cells. In this study, we systematically investigated the nucleosome occupancy profiles around TFBSs and their relevance to gene expression in human GM12878 and K562 cells. The nucleosomes were generally depleted at TFBSs *in vivo*, and asymmetric patterns of nucleosome occupancy were more pervasive around TFBSs. However, approximately a quarter of TFBSs showed well-positioned nucleosomes on both sides, and a small proportion of TFBSs were occupied by nucleosomes. Compared with the distal sites, proximal sites showed fuzzier nucleosome positioning. These proximal sites were located within 1 kb of TSSs, and RNA polymerase complexes near the TSSs had a large effect on the nucleosome occupancy distributions around these sites. Compared with activator sites, nucleosomes around repressor sites were better positioned. In addition, nucleosome occupancy patterns around TFBSs were correlated with the expression levels of target genes. Genes with repressor sites exhibiting well-positioned nucleosomes on both sides, and genes with activator sites occupied by nucleosomes had significantly lower expression.

## Methods

### Data source

The aligned MNase-seq reads for *in vivo* nucleosomes in both GM12878 and K562 cells [29], were generated by the Snyder lab and downloaded from University of California Santa Cruz (UCSC) Genome Browser FTP server (ftp://hgdownload.soe.ucsc.edu/goldenPath/hg19/encodeDCC/). Downloaded files contained reads mapped to the hg19 human reference genome in the BAM format, and we therefore converted these to BED format using BEDTools [35]. We further removed duplicate reads that were exactly mapped to the same position, as these reads might arise from biases during ChIP-DNA amplification and sequencing library preparation [36]. In order to determine the *in vivo* nucleosome occupancy level at each genomic coordinate, we shifted the start position of each read by 73 bp in the 5′ to 3′ direction, and counted the total number of reads with a window size of 60 bp on both strands. These counts were finally normalized by the expected number of reads in the 60-bp window, which was calculated as:

The *in vitro* nucleosomes were assembled through combining the human genomic DNA with recombinantly derived histone octamers [13], and the raw sequenced reads for *in vitro* nucleosomes were obtained from the NCBI Gene Expression Omnibus (http://www.ncbi.nlm.nih.gov/geo/) using the accession number GSE25133. The sequenced reads were first mapped to the hg19 human genome using the Bowtie aligner [37], allowing a maximum of two mismatches. Then duplicate mapped reads were removed and the rest of reads were shifted by 73 bp in the 5′ to 3′ direction. Reads within a 60-bp window were finally counted and normalized to construct the *in vitro* nucleosome occupancy profile along the human genome, as in the processing of *in vivo* nucleosome reads.

The peaks of enriched signals in DNase I hypersensitivity experiments [38], generated by the Crawford lab, were downloaded from the UCSC FTP server. Crawford’s group mapped DNase-seq reads to the hg19 human genome using the BWA aligner [39], calculated the signal enrichment at each genomic coordinate using the F-Seq software [40], and identified peaks from the F-Seq density signals. These DNase I-hypersensitive regions reflected the openness of the chromatin and the accessibility of the genome in GM12878 and K562 cells.

TF binding sites for 519 binding motifs estimated with CENTIPEDE [19] were downloaded from http://centipede.uchicago.edu/SimpleMulti/. The total numbers of binding sites in the GM12878 and K562 cells were 368,127 and 340,094, respectively. The initial downloaded data were mapped to the hg18 human reference genome, and the binding locations were therefore converted from hg18 to hg19 using liftOver, provided by the UCSC Genome Browser. CENTIPEDE scanned the human genome with a TF binding motif to obtain candidate binding sites and computed a posterior probability for each candidate site to identify the real binding sites. It should be noted that although CENTIPEDE predicted binding sites using 519 TF binding motifs, only 220 and 260 binding motifs (Additional file 10) were included in the GM12878 and K562 cells, respectively, to ensure that each of the binding sites in downloaded files had a posterior probability greater than 0.999.

The aligned RNA-seq reads in GM12878 and K562 cells [41], were generated by the Caltech and downloaded from the UCSC FTP server. These paired-end reads were aligned to the hg19 genome and stored in the BAM format. The hg19 RefSeq gene annotation data [42] were also obtained from the UCSC FTP server. In order to determine the TSS position and the expression level of each of the genes, we first removed non-protein-coding transcripts from the hg19 RefSeq file. Then, we used the transcript assembly and quantification software Cufflinks [43] with default settings to calculate the expression value of each RefSeq transcript, which was quantified in fragments per kilobase of exon per million mapped fragments [44]. For alternatively spliced transcripts encoding the same protein, only the transcript with the highest expression value was used. A total of 19,019 TSSs of RefSeq genes in both GM12878 and K562 cells were obtained to investigate the nucleosome occupancy around TSSs and define the distance between a binding site and the nearest TSS. We further classified genes into four categories on the basis of their expression levels. Genes with expression levels less than the first quartile, between the first and second quartiles, between the second and third quartiles, and greater than the third quartile were considered as very lowly, lowly, medium and highly expressed genes, respectively.

### Nucleosome occupancy around TF binding sites

For a group of CENTIPEDE sites, we extracted the nucleosome signal in a ±2-kb window around each binding site and averaged nucleosome signals over all sites to represent the nucleosome occupancy around the binding sites. In addition, considering the confounding factors of nearby TSSs, we assigned each of the binding sites to the nearest gene based on its distance to the TSSs and reversed the shape profile of binding sites on the negative strand before averaging, to avoid a misleading aggregation.

### Identification of activators and repressors

All activators and repressors were first retrieved from the UniProt database [26], a comprehensive resource for protein sequence and annotation data, to determine whether a TF was an activator or repressor. The search terms for activators and repressors were “activator AND organism:human AND reviewed:yes” and “repressor AND organism:human AND reviewed:yes”, respectively. Some multifunctional TFs, such as YY1 and CTCF, were annotated as both activators and repressors and were further removed in the analysis. We finally identified 20 activators and four repressors in the GM12878 cell line, and 25 activators and six repressors in the K562 cell line (Additional file 2).

### Clustering nucleosome occupancy around TF binding sites

We extracted the nucleosome signals in a ±500-bp window around each binding site, and clustered these nucleosome signals using the CAGT software [29]. CAGT uses the *k*-medians algorithm to obtain a relatively large number of compact clusters, and then redundant clusters are merged using the hierarchical agglomerative clustering. The number of clusters was set to 40 and a correlation-based distance function was used in the *k*-medians clustering in our analyses. Hierarchical agglomerative clustering iteratively merged the two most similar clusters and mirror clusters were also merged. If the number of clusters was set to 1, a distance threshold was set to 0.4 and two closest clusters with a distance below the threshold would be merged in the hierarchical agglomerative clustering. In addition, binding sites, whose nucleosome signal profiles had variance below a threshold of 0.1, were removed prior to the *k*-medians clustering.

## Electronic supplementary material

Additional file 1: **519 TF binding motifs and their corresponding TFs.** The list included 519 TF binding motifs and their corresponding TFs. One TF may have more than one binding motifs. (XLSX 22 KB)

Additional file 2: **Activators and repressors in GM12878 and K562 cells.** The list included 20 activators and four repressors in GM12878 cells, and 25 activators and six repressors in K562 cells, which were identified by querying the UniProt database. (XLSX 10 KB)

Additional file 3: **Coordinates of the distal activator and repressor binding sites in GM12878 and K562 cells.** The four sheets contained the coordinates of 24,133 distal activator sites and 3,422 distal repressor sites in the GM12878 cell line, and 13,840 distal activator sites and 2,489 distal repressor sites in the K562 cell line. (XLSX 2 MB)

Additional file 4: ***In vivo*** **nucleosome occupancy around the distal binding sites for each of activators.** (PDF 791 KB)

Additional file 5: ***In vivo*** **nucleosome occupancy around the distal binding sites for each of repressors.** (PDF 188 KB)

Additional file 6: **Clustering nucleosome signals around proximal and distal binding sites.** **(A, B)** Nucleosome occupancy clusters around proximal sites in GM12878 and K562 cells. **(C, D)** Nucleosome occupancy clusters around distal sites in GM12878 and K562 cells. (PDF 668 KB)

Additional file 7: **Clustering nucleosome signals around distal activator and repressor binding sites.** **(A, B)** Nucleosome occupancy clusters around distal activator sites in GM12878 and K562 cells. **(C, D)** Nucleosome occupancy clusters around distal repressor sites in GM12878 and K562 cells. (PDF 539 KB)

Additional file 8: **Three nucleosome occupancy clusters around distal activator and repressor binding sites.** **(A, B)** Three nucleosome occupancy clusters for distal activator binding sites in GM12878 and K562 cells. **(C, D)** Three nucleosome occupancy clusters for distal repressor binding sites in GM12878 and K562 cells. (PDF 420 KB)

Additional file 9: **Expression levels of target genes for each cluster of distal activator and repressor binding sites.** **(A, B)** Expression levels of target genes for each cluster of distal activator binding sites in GM12878 and K562 cells. **(C, D)** Expression levels of target genes for each cluster of distal repressor binding sites in GM12878 and K562 cells. (PDF 10 KB)

Additional file 10: **TF binding motifs in GM12878 and K562 cells.** The list included 220 motifs in the GM12878 cell line and 260 motifs in the K562 cell line. Each of the binding sites for these motifs had a posterior probability greater than 0.999. (XLSX 18 KB)

## Abbreviations

TF: Transcription factor
TFBS: Transcription factor binding site
TSS: Transcription start site
CAGT: Clustered AGgregation tool.
