# Supplementary figures and images for: Nucleosome organization in the vicinity of transcription factor binding sites in the human genome
Source: BMC Genomics. 2014 Jun 19;15(1):493. doi: 10.1186/1471-2164-15-493 (PMC4073502; doi:10.1186/1471-2164-15-493)

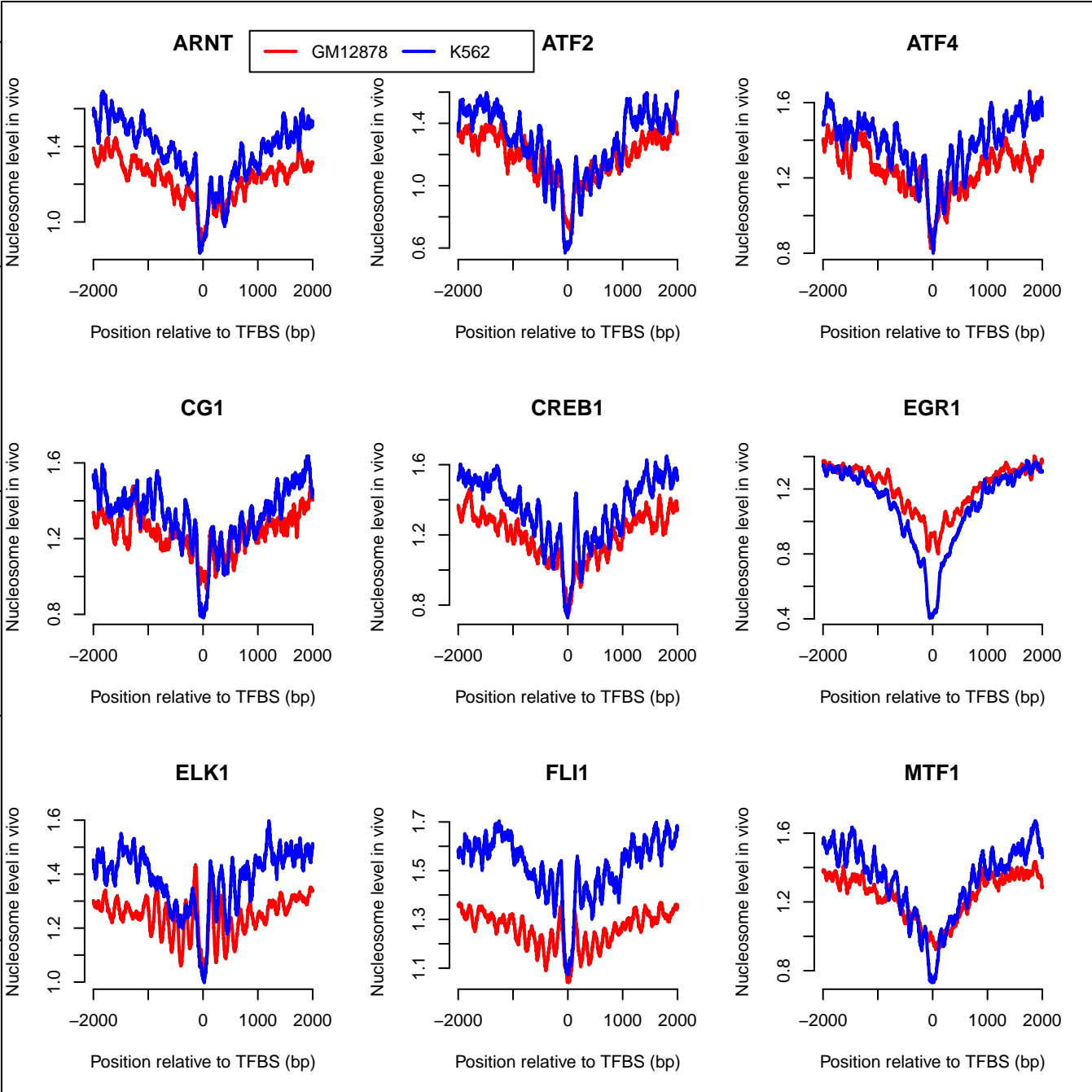

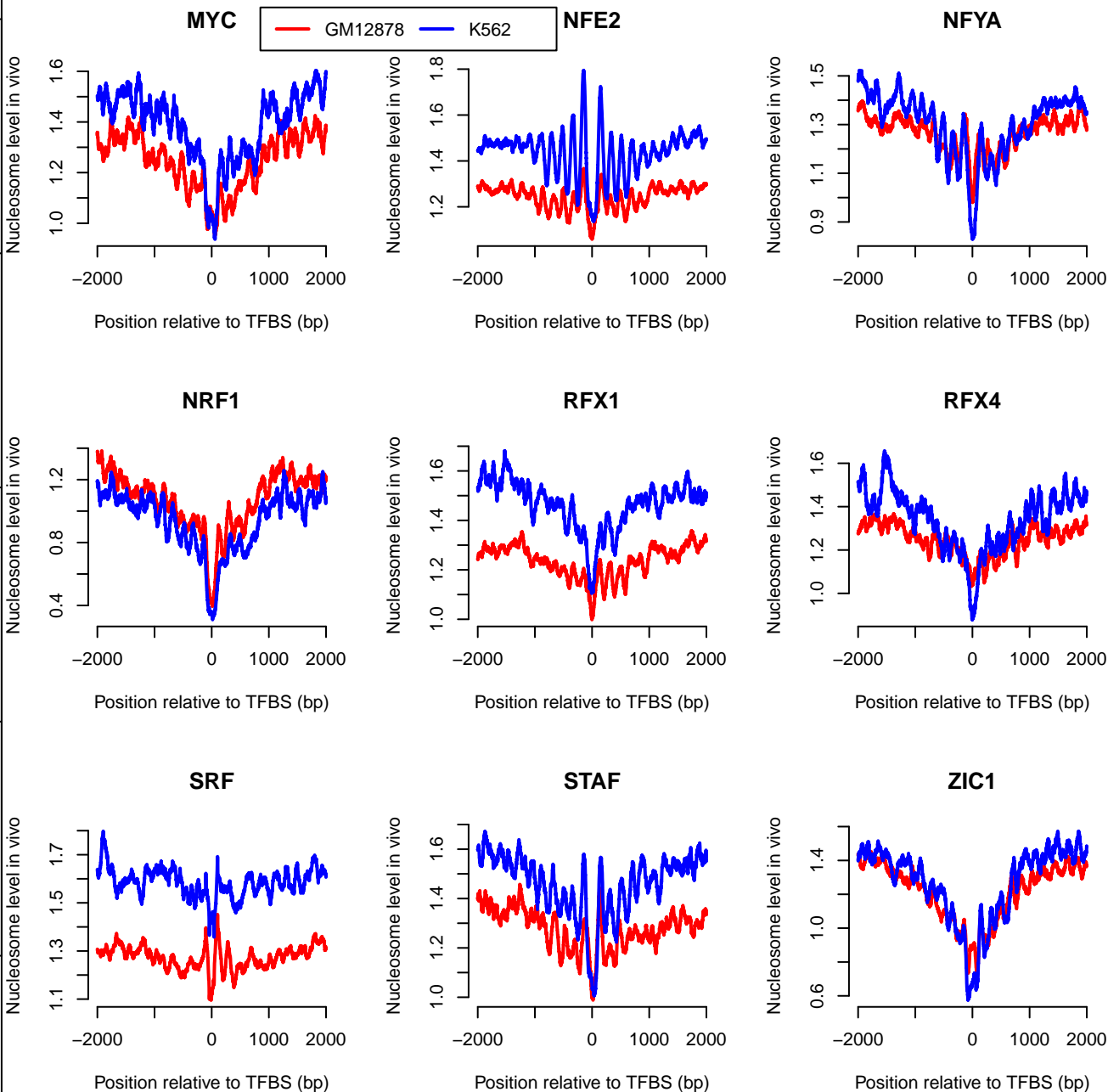

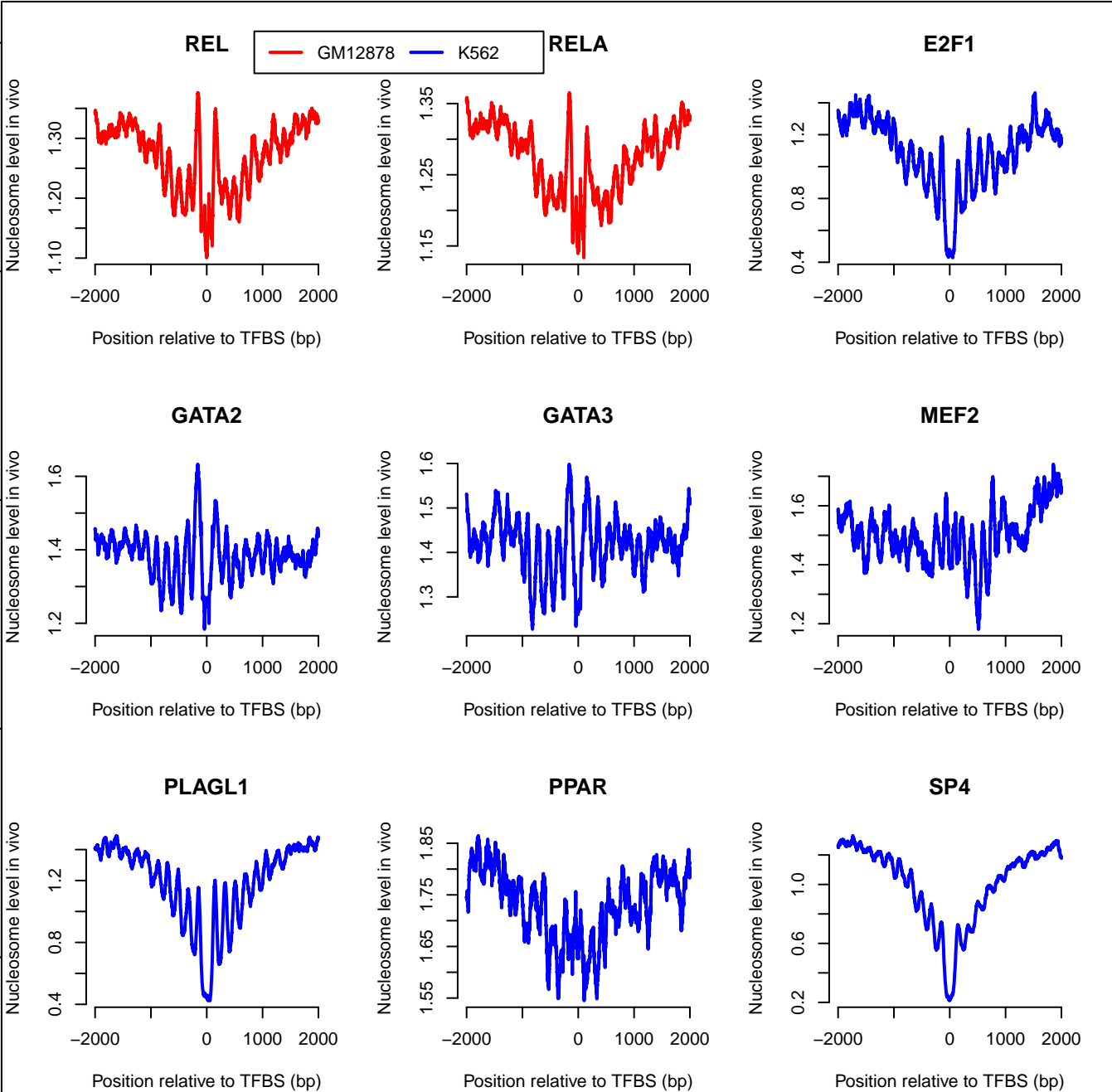

Supplement: Supplementary file 4 — Additional file 4: In vivo nucleosome occupancy around the distal binding sites for each of activators. (PDF 791 KB) [file 12864_2013_6160_MOESM4_ESM.pdf]

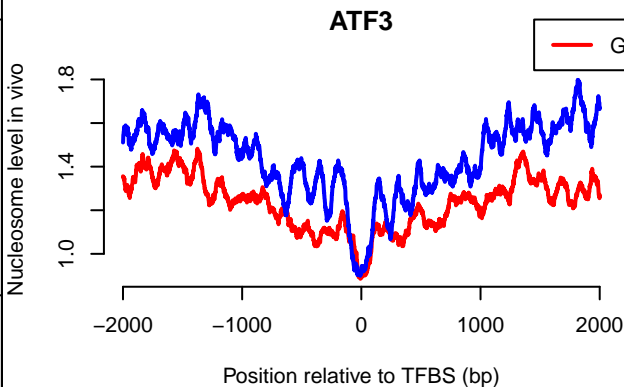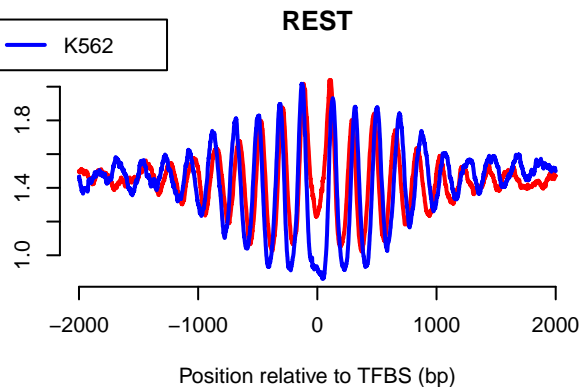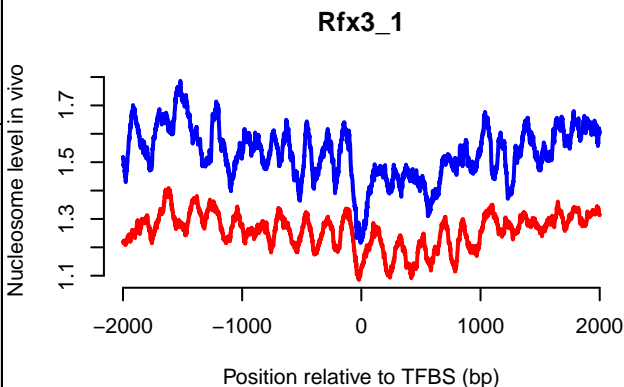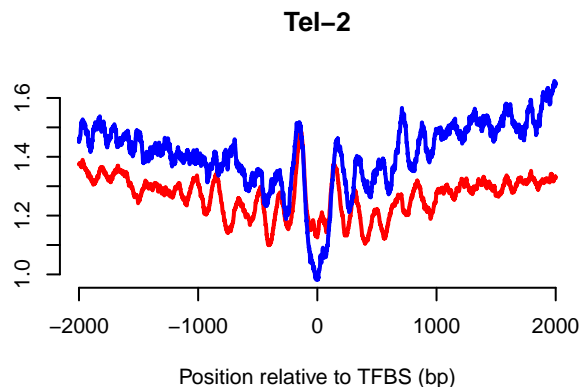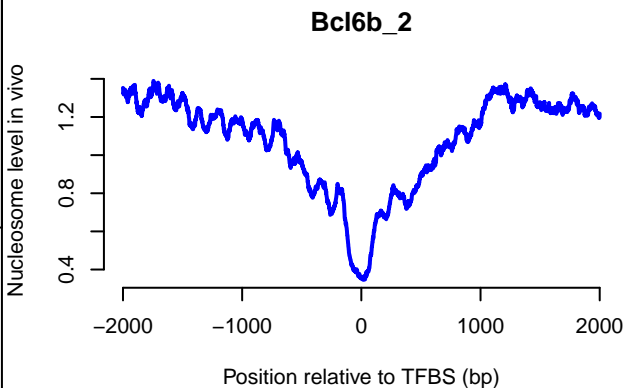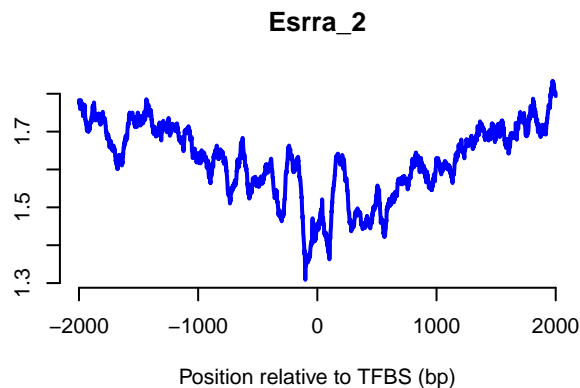

Supplement: Supplementary file 5 — Additional file 5: In vivo nucleosome occupancy around the distal binding sites for each of repressors. (PDF 188 KB) [file 12864_2013_6160_MOESM5_ESM.pdf]

## A Proximal sites in GM12878

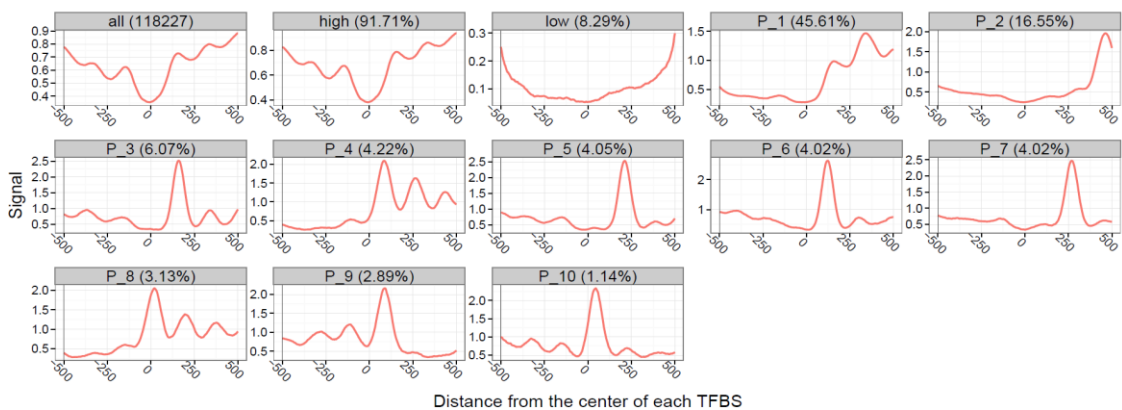

## B Proximal sites in K562

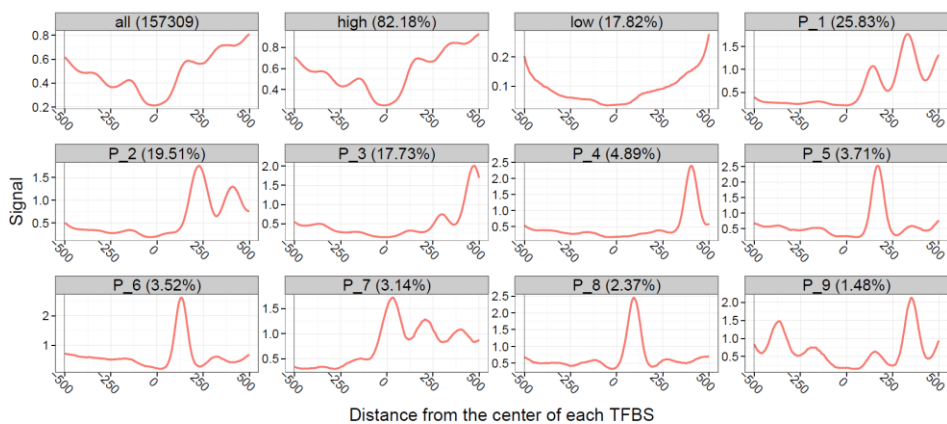

## C Distal sites in GM12878

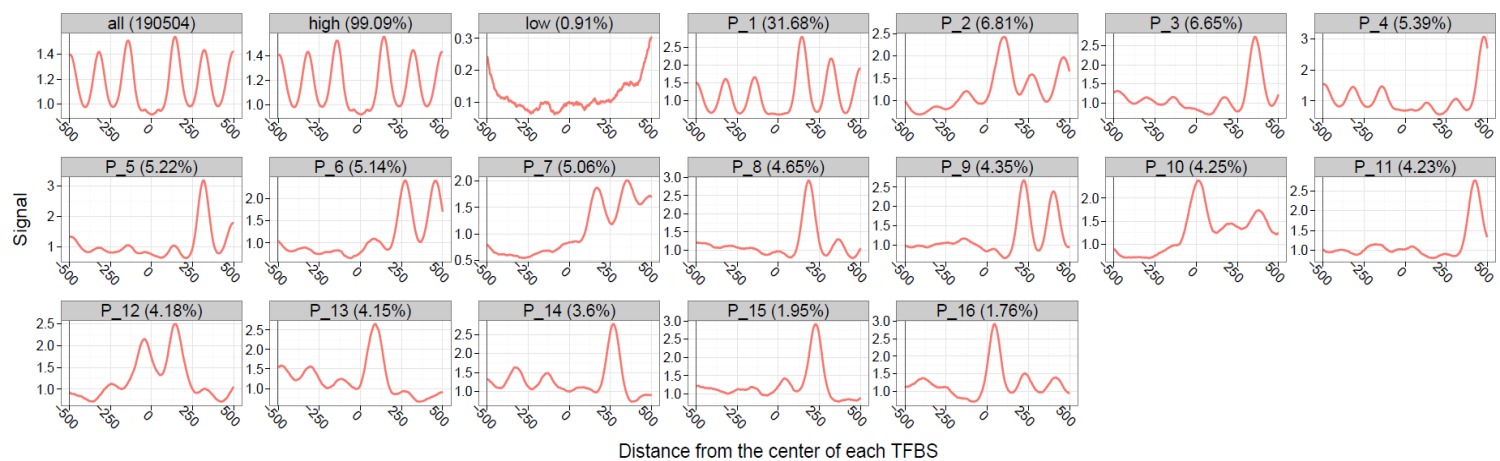

## D Distal sites in K562

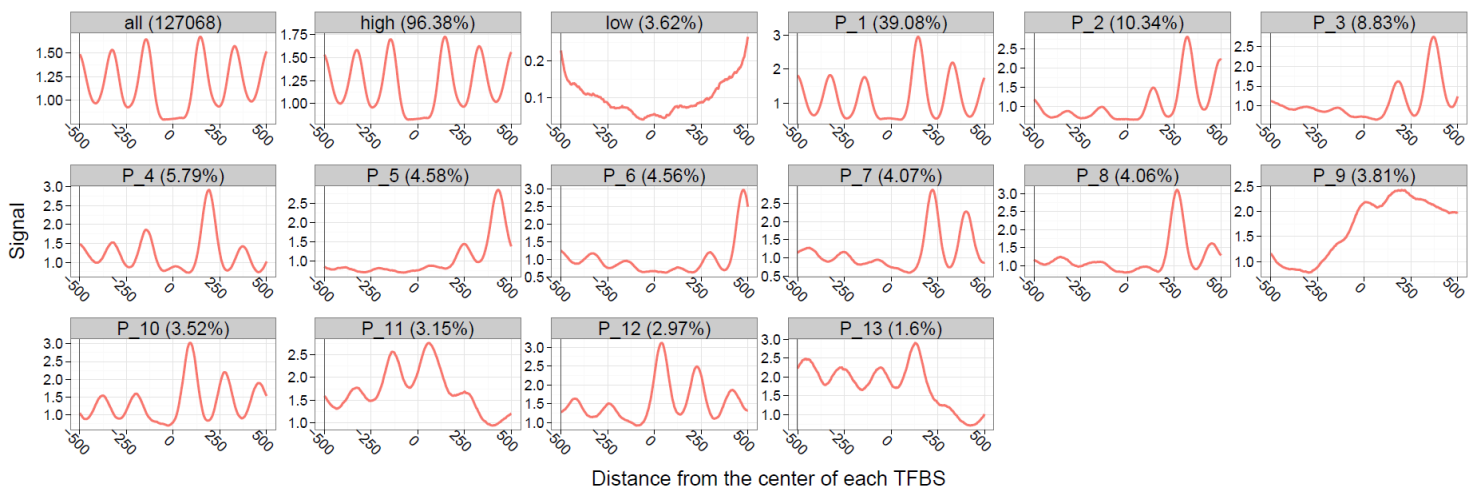

Supplement: Supplementary file 6 — Additional file 6: Clustering nucleosome signals around proximal and distal binding sites. (A, B) Nucleosome occupancy clusters around proximal sites in GM12878 and K562 cells. (C, D) Nucleosome occupancy clusters around distal sites in GM12878 and K562 cells. (PDF 668 KB) [file 12864_2013_6160_MOESM6_ESM.pdf]

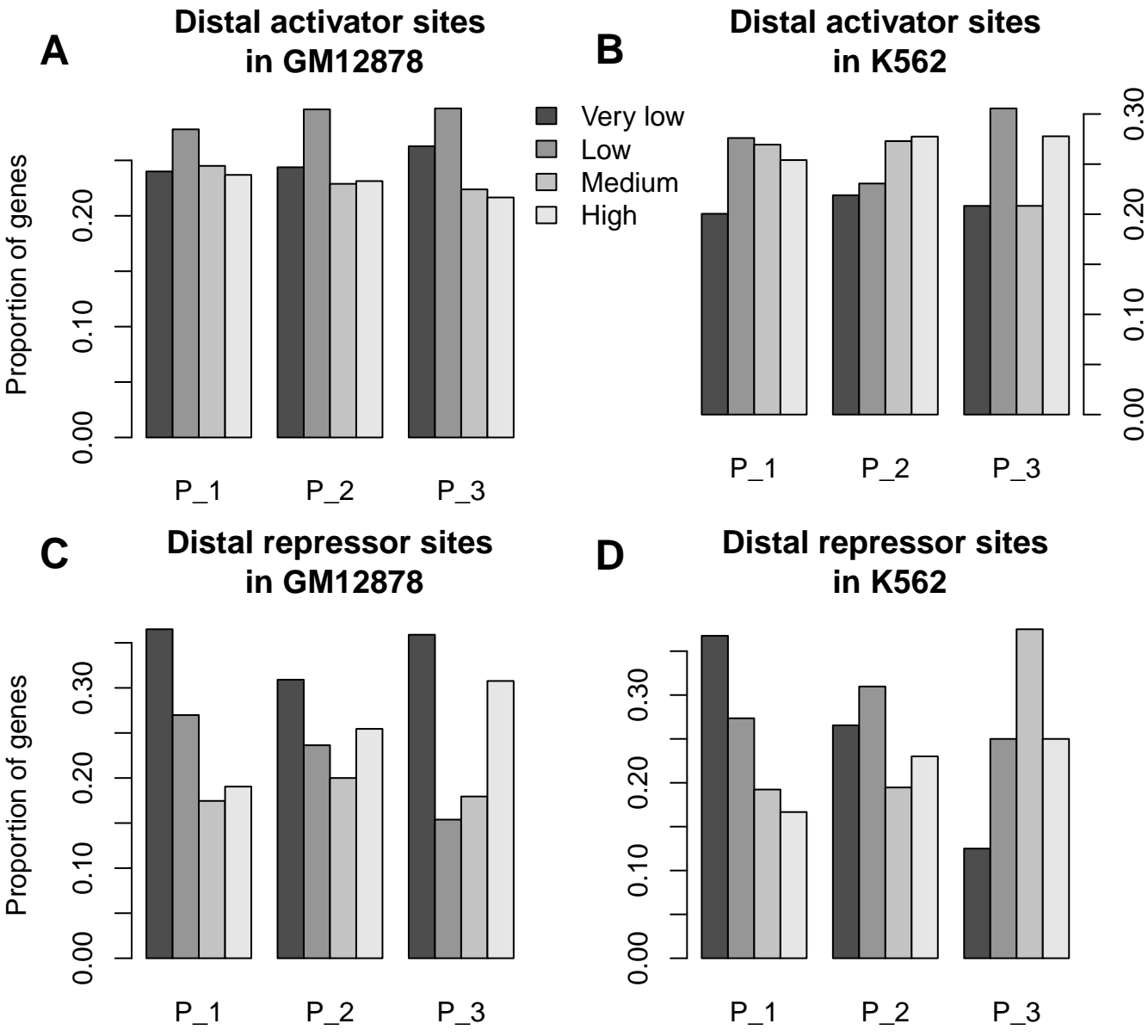

Supplement: Supplementary file 9 — Additional file 9: Expression levels of target genes for each cluster of distal activator and repressor binding sites. (A, B) Expression levels of target genes for each cluster of distal activator binding sites in GM12878 and K562 cells. (C, D) Expression levels of target genes for each cluster of distal repressor binding sites in GM12878 and K562 cells. (PDF 10 KB) [file 12864_2013_6160_MOESM9_ESM.pdf]
