# Supplementary material for: Nucleosome organization in the vicinity of transcription factor binding sites in the human genome
Source: BMC Genomics. 2014 Jun 19;15(1):493. doi: 10.1186/1471-2164-15-493 (PMC4073502; doi:10.1186/1471-2164-15-493)

A Distal activator sites in GM12878

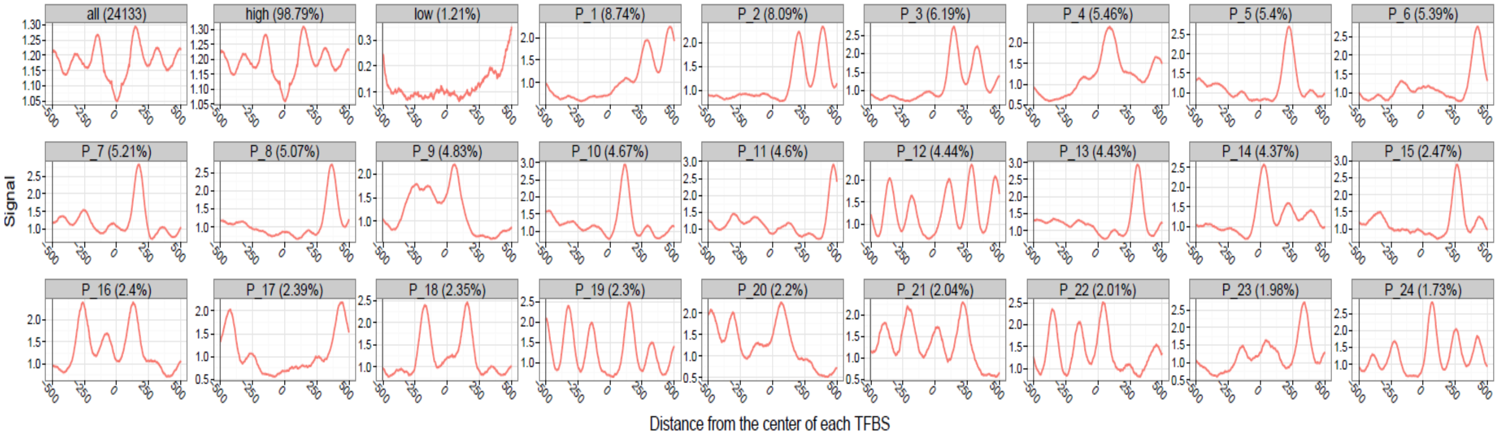

B Distal activator sites in K562

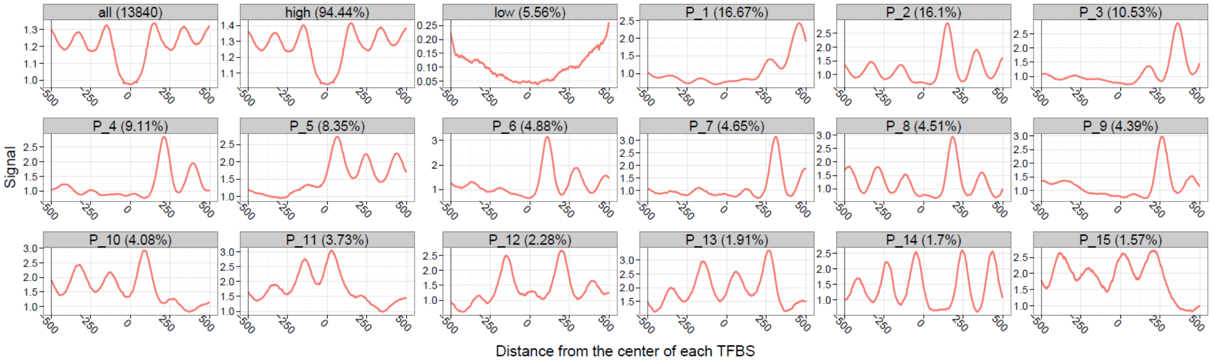

C Distal repressor sites in GM12878

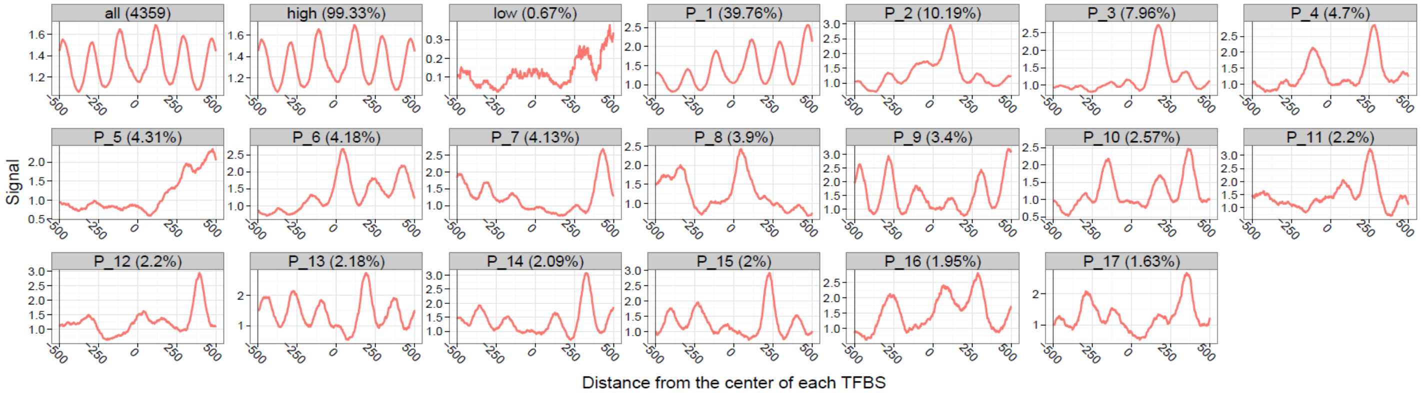

D Distal repressor sites in K562

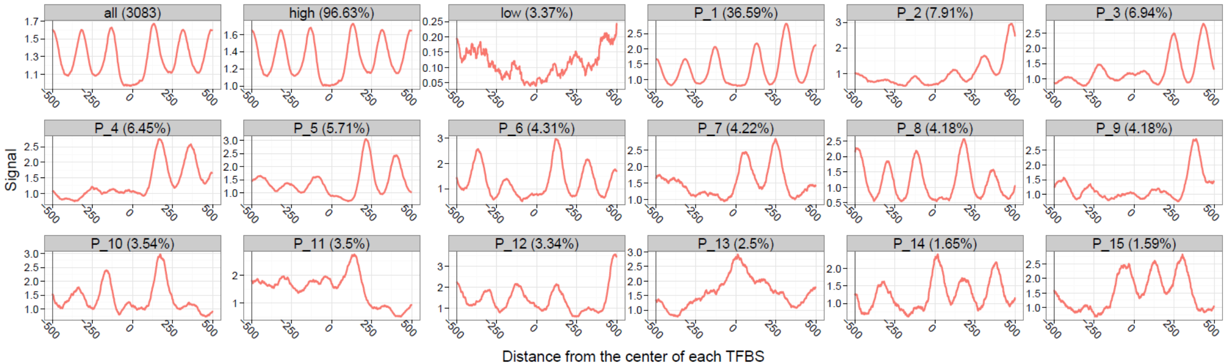

Supplement: Supplementary file 7 — Additional file 7: Clustering nucleosome signals around distal activator and repressor binding sites. (A, B) Nucleosome occupancy clusters around distal activator sites in GM12878 and K562 cells. (C, D) Nucleosome occupancy clusters around distal repressor sites in GM12878 and K562 cells. (PDF 539 KB) [file 12864_2013_6160_MOESM7_ESM.pdf]
