# Supplementary material for: Nucleosome organization in the vicinity of transcription factor binding sites in the human genome
Source: BMC Genomics. 2014 Jun 19;15(1):493. doi: 10.1186/1471-2164-15-493 (PMC4073502; doi:10.1186/1471-2164-15-493)
Supplement: Supplementary file 8 — Additional file 8: Three nucleosome occupancy clusters around distal activator and repressor binding sites. (A, B) Three nucleosome occupancy clusters for distal activator binding sites in GM12878 and K562 cells. (C, D) Three nucleosome occupancy clusters for distal repressor binding sites in GM12878 and K562 cells. (PDF 420 KB) [file 12864_2013_6160_MOESM8_ESM.pdf]

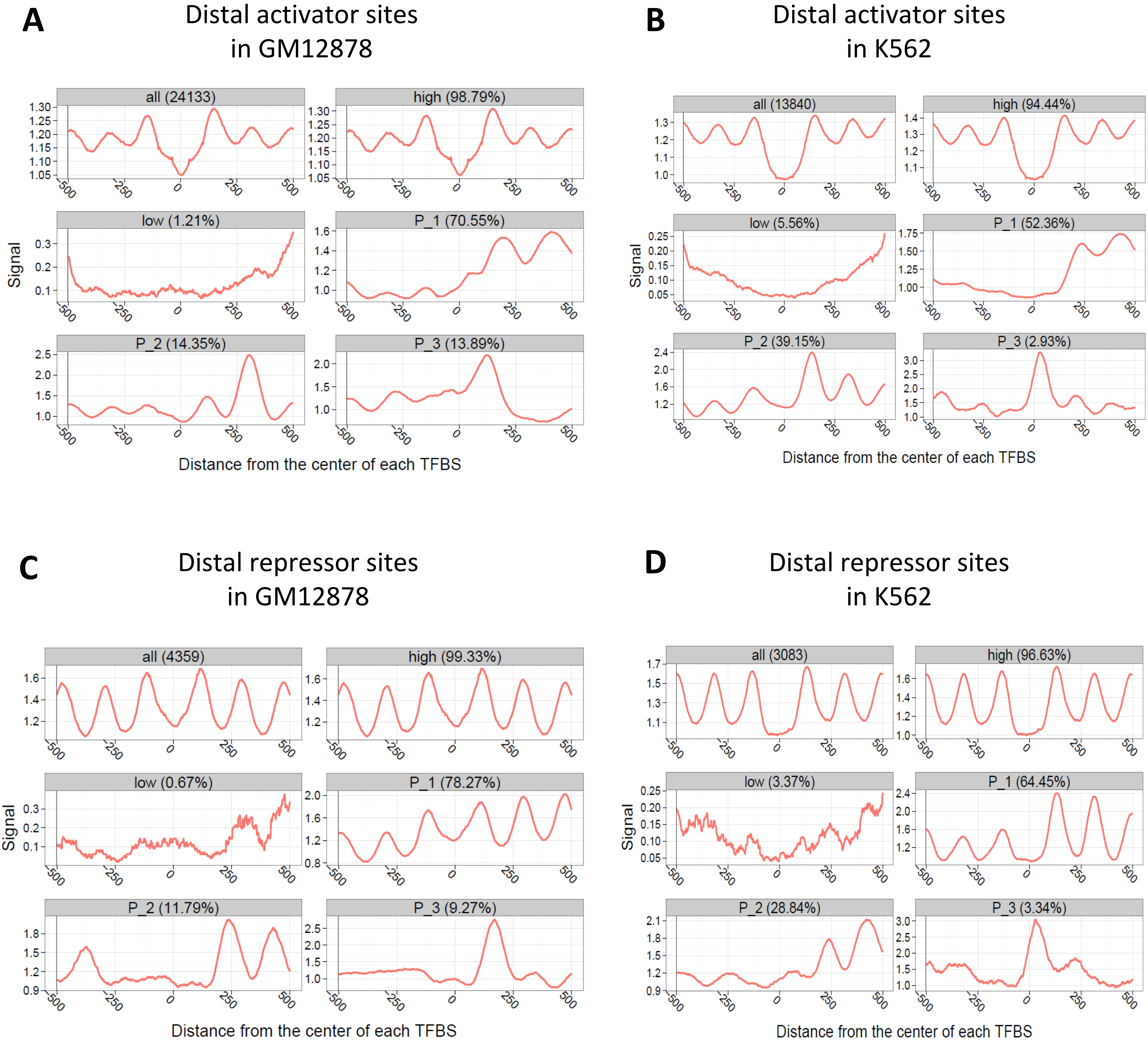
A
Distal activator sites
in GM12878
all (24133)
high (98.79%)
low (1.21%)
P_1 (70.55%)
P_2 (14.35%)
P_3 (13.89%)
Signal
Distance from the center of each TFBS
B
Distal activator sites
in K562
all (13840)
high (94.44%)
low (5.56%)
P_1 (52.36%)
P_2 (39.15%)
P_3 (2.93%)
Signal
Distance from the center of each TFBS
C
Distal repressor sites
in GM12878
all (4359)
high (99.33%)
low (0.67%)
P_1 (78.27%)
P_2 (11.79%)
P_3 (9.27%)
Signal
Distance from the center of each TFBS
D
Distal repressor sites
in K562
all (3083)
high (96.63%)
low (3.37%)
P_1 (64.45%)
P_2 (28.84%)
P_3 (3.34%)
Signal
Distance from the center of each TFBS
